# Ocular manifestations and immunological profiles of thyroid eye disease with lacrimal gland enlargement

**DOI:** 10.3389/fendo.2026.1861245

**Published:** 2026-07-15

**Authors:** Yan Sun, Fan Shi, Congyao Wang, Xia Dong, Yihua Su, Fenfen Yu, Shubin Hong, Dide Wu, Mengsha Zou, Zhiyun Yang, Haipeng Xiao, Pengxia Wan

**Affiliations:** 1 Department of Ophthalmology, The First Affiliated Hospital, Sun Yat-sen University, Guangzhou, Guangdong, China; 2 Department of Endocrinology, The First Affiliated Hospital, Sun Yat-sen University, Guangzhou, Guangdong, China; 3 Department of Radiology, The First Affiliated Hospital, Sun Yat-sen University, Guangzhou, Guangdong, China

**Keywords:** lacrimal gland enlargement, T cell subset, thyroid eye disease, thyroid peroxidase antibody, Treg cell

## Abstract

**Introduction:**

Thyroid eye disease (TED) is a prevalent autoimmune inflammatory condition in patients diagnosed with Graves’ disease (GD). The lacrimal glands are implicated as an additional target organ of autoimmune reactions in TED, alongside orbital connective tissues. However, the underlying pathogenesis of lacrimal gland enlargement in TED and its associated symptoms and effective therapeutic strategies remain to be elucidated. This study aimed to investigate the ocular manifestations and immunological profiles of TED with lacrimal gland enlargement.

**Methods:**

This study collected patients diagnosed with TED, divided into the enlarged lacrimal gland (ELG) and the normal lacrimal gland (NLG) groups based on magnetic resonance imaging (MRI) measurement. Ocular manifestations, thyroid function tests, and immunological examinations of the peripheral blood were systematically compared between the two groups.

**Results:**

123 TED patients (NLG: ELG = 36: 87) were enrolled in this study. Patients in the ELG group exhibited worse proptosis, decreased tear film break-up time (TBUT), and increased Schirmer I test values, compared to the NLG group (P < 0.05), with no statistical difference in clinical activity score (CAS). Lacrimal gland enlargement in TED was accompanied by an elevation in thyroid peroxidase antibody (TPO-Ab) levels. Moreover, the proportions of CD8^+^ T cells and regulatory T (Treg) cells were remarkably elevated in the ELG group (P < 0.05). Correlation analyses demonstrated that serum TPO-Ab levels and Treg cell proportions were significantly correlated with lacrimal gland area. Further multivariate regression analyses confirmed that both TPO-Ab and Treg cells served as independent influencing factors for lacrimal gland enlargement in patients with TED.

**Conclusions:**

TED patients with enlarged lacrimal glands exhibited worse proptosis, unstable tear film, and excessive tearing. TPO-Ab and Treg cells were positively correlated with lacrimal gland enlargement in TED. Furthermore, Treg cell frequency emerged as a predictive biomarker for lacrimal gland involvement in TED patients.

## 1 Introduction

Thyroid eye disease (TED), also known as Graves’ ophthalmopathy (GO), serves as the predominant extrathyroidal manifestation of Graves’ disease (GD) (1, 2). Patients afflicted with TED endure a spectrum of clinical manifestations, encompassing periorbital edema, eyelid retraction, proptosis, and a host of other distressing symptoms (3). The pathogenesis of TED involves inflammatory alterations within the orbital adipose tissue, extraocular muscles, and notably, the lacrimal glands (4). A remarkable characteristic of TED is the expression of thyroid-stimulating hormone receptors (TSHR) in the lacrimal glands of nearly all affected patients, demonstrating histological similarity to the thyroid gland and rendering it susceptible to immune-mediated injury (5, 6). The lacrimal gland, strategically positioned in the superior temporal orbit, plays a pivotal role in synthesizing the aqueous component of the tear film, thereby maintaining ocular surface integrity and visual acuity (7). Consequently, the direct involvement of the lacrimal gland in TED precipitates ocular surface damage, exacerbating the clinical burden of the disease (8). Relevant studies have increasingly demonstrated a significant prevalence of lacrimal gland involvement in patients with TED (9, 10).

Imaging studies using computed tomography (CT) and magnetic resonance imaging (MRI), have quantitatively evaluated significant enlargement of lacrimal gland volume in patients with TED (11–13). Several studies revealed that patients with lacrimal gland enlargement had a higher proportion of active TED (9, 14), subjective tearing (15, 16), and worse proptosis (15, 17). Despite these findings, a comprehensive quantitative assessment of the ocular manifestations stemming from lacrimal gland enlargement in TED remains conspicuously absent.

Numerous diseases that can result in lacrimal gland enlargement, including IgG4-related disease and sickle cell disease (18, 19), are intricately linked to immune-related factors (20). The previous pathological observation of the lacrimal glands in active TED indicates that this involvement is characterized by lymphocyte infiltration and interstitial edema (21). These findings suggested that immune responses targeting the lacrimal gland might contribute to an inflammatory process and is linked to edema of the lacrimal gland (22). However, the underlying etiology of lacrimal gland enlargement during TED progression remains elusive, and conclusive evidence regarding the efficacy of treatments for alleviating TED-induced lacrimal gland enlargement is scarce.

Our previous study has demonstrated that multi-parametric MRI of the lacrimal gland exhibits exceptional predictive capabilities for diagnosing and staging TED (12). Upon this foundation, we categorized TED patients into the normal lacrimal gland (NLG) and enlarged lacrimal glands (ELG) groups based on MRI measurements. In this study, ocular symptoms and parameters were compared between the two groups. Moreover, we investigated the associations between enlarged lacrimal glands and serum cytokines, thyroid function, peripheral lymphocyte subgroups, and T cell functional subsets. The objective of this study was to deepen our understanding of lacrimal gland enlargement in TED, providing a crucial reference for unraveling its pathogenesis and identifying potential therapeutic approaches that could improve patient outcomes in the future.

## 2 Methods

### 2.1 Study design and participants

This cross-sectional study consecutively enrolled patients who were initially diagnosed with TED who had not received systemic glucocorticoids or immunosuppressants treatment, at the First Affiliated Hospital of Sun Yat-sen University, between 2019 and 2024. The study was conducted in accordance with the Declaration of Helsinki and received approval from the Ethics Committee of the First Affiliated Hospital of Sun Yat-sen University. The inclusion criteria were as follows: (1) diagnosis of TED according to Bartley’s diagnostic criteria (23); (2) adequate quality structural orbital MRI for the measurement of the lacrimal gland. The exclusion criteria were as follows: (1) history of orbital radiotherapy or ocular surgery or ocular trauma; (2) unilateral lacrimal gland enlargement; (3) other local or systemic diseases known to affect the lacrimal gland size [e.g., IgG4-related disease, idiopathic orbital inflammatory disease, rheumatologic or inflammatory diseases (20, 24)]; (4) current pregnancy or lactation; (5) history of psychosis, significant comorbidities (e.g., cardiovascular disease, poorly managed diabetes mellitus, malignant hypertension), and cancers. Demographic characteristics including age and gender were recorded on the first clinical visit. The same experienced ophthalmologist performed ophthalmologic examinations. The First Affiliated Hospital of Sun Yat-sen University laboratory department completed all blood examinations. All enrolled participants provided written informed consent before the examination.

### 2.2 MRI acquisition and processing

**Figure 1:**
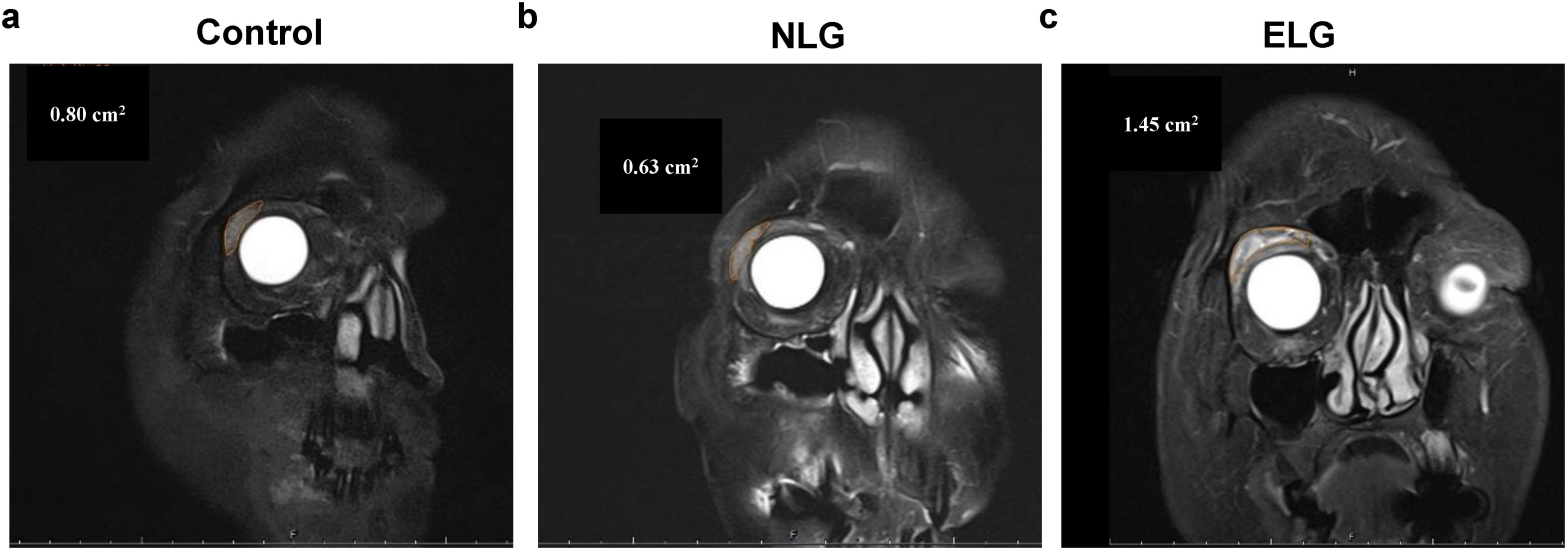
MRI scans in oblique coronal T2WI with the Dixon sequence of the lacrimal gland. The image in which the lacrimal gland appeared the largest in oblique coronal T2WI with the Dixon sequence was chosen and the area of the lacrimal gland was measured by manually delineating the gland border. **(a)** A 57-year-old control patient without TED, area = 0.80 cm^2^. **(b)** A 59-year-old TED patient in the NLG group, area = 0.63 cm^2^. **(c)** A 56-year-old TED patient in the ELG group, area = 1.45 cm^2^.

MRI examinations were performed on a 3.0 T MRI system (Prisma, Siemens Healthcare, Erlangen, Germany) with a 32-channel brain coil. Imaging sequences comprised axial T1 weighted images (WI), T2WI, and oblique coronal T2WI with Dixon sequence. MRI measurements of bilateral lacrimal glands were conducted on oblique coronal T2WI images with the Dixon sequence for all included patients. Due to the indistinguishability of palpebral and orbital lobes on MRI, the lacrimal gland was considered as one structure. Lacrimal gland enlargement was evaluated on orbital imaging examinations by two senior radiologists who were blinded to the clinical information. The average of three repeated measurements of the maximum area of the lacrimal gland in the oblique coronal position of the right eye for each patient was used in further statistical analysis (**Figure 1**). We defined lacrimal gland enlargement as the maximum area of the lacrimal gland in the oblique coronal position > 1 cm², which was established with reference to the mean value of the normal population. If there was ambiguity in the grouping after measurement, it would be decided by a third radiologist.

### 2.3 Ocular evaluations

Clinical activity score (CAS) was evaluated based on the following seven items: pain on attempted up or down gaze, spontaneous retrobulbar pain, swelling of the eyelids, redness of the conjunctiva, redness of the eyelids, chemosis, and swollen caruncle (25). The evaluation of CAS was performed separately for each eye, and patient classification was based on the higher score obtained from either eye. Following assessment by an experienced endocrinologist, eyes with a CAS of ≥ 3/7 were classified into the active stage group; otherwise, they were classified into the inactive stage group. The severity of TED was evaluated using the European Group on Graves Ophthalmopathy (EUGOGO) criteria (26). A Hertel exophthalmometer was used to measure the degree of ocular proptosis. Tear film break-up time (TBUT) was measured to assess the tear film stability. TBUT less than 10 seconds was indicative of unstable tear film. Schirmer I test without anesthesia was performed for each patient. The average of three successive measurements was used in the analysis.

### 2.4 Thyroid function tests

Serum samples were collected from the enrolled subjects for subsequent analyses in the hospital clinical laboratory. Thyroid-related biochemical parameters, including TSH, free triiodothyronine (FT3), total triiodothyronine (TT3), free thyroxine (FT4), total thyroxine (TT4), thyroglobulin (TG), anti-thyroglobulin antibody (TG-Ab), thyroid peroxidase antibody (TPO-Ab), and TSH receptor antibody (TR-Ab), were quantified on an automated chemiluminescence analyzer (DXI 800, Beckman Coulter, USA). All biochemical measurements were performed in strict compliance with the manufacturer’s instructions.

### 2.5 Serum cytokines

The serum levels of interleukin-2 (IL-2), interleukin-4 (IL-4), interleukin-6 (IL-6), interleukin-10 (IL-10), tumor necrosis factor-α (TNF-α), and interferon-γ (IFN-γ) were quantified using commercial enzyme-linked immunosorbent assay (ELISA) kits. All ELISA kits were purchased from R&D Systems (Minneapolis, MN, USA) and all experimental operations were carried out in strict accordance with the kit manufacturer’s protocols. The absorbance value of each well was measured at a wavelength of 450 nm using a microplate reader (Bio-Rad, CA, USA) after the termination of the chromogenic reaction. Each sample was equipped with duplicate wells to reduce experimental error, and the average value was taken as the final detection result.

### 2.6 Peripheral lymphocyte subgroups and T cell subsets

Peripheral venous blood samples were collected from participants using EDTA as an anticoagulant. Red blood cells were lysed with lysing solution buffer. Flow cytometric analyses were performed on a BD Canto flow cytometer. Raw flow cytometry data were processed and analyzed via Kaluza software. Detailed staining procedures for the immunophenotyping of lymphocytes and T cell subsets were described below.

A multicolor antibody panel for peripheral lymphocyte subpopulations included CD3 PB, CD4 APC-Cy7, CD8 PE-Cy7, CD19 PE, CD16 FITC, CD56 APC, and CD45 KO. Leukocytes were gated by CD45 expression versus side scatter (SSC), and CD3^+^ T lymphocytes were subsequently identified and subdivided into CD3^+^CD4^+^ and CD3^+^CD8^+^ subsets. The proportions of major lymphocyte subsets, including CD3^+^ total T cells, CD4^+^ helper T cells, CD8^+^ T cells, CD19^+^ B cells, and CD3^−^CD16^+^CD56^+^ natural killer (NK) cells, together with the CD4^+^/CD8^+^ ratio, were quantified.

A second multicolor antibody panel of T cell subsets included CD3 PB, CD4 APC-Cy7, CD8 PE-Cy7, CD28 PE, CD38 APC, HLA-DR FITC, PD-1 PerCP-Cy7, and CD45 KO. The expression of T cell activation markers (CD28, CD38, HLA-DR) and the inhibitory marker PD-1 was analyzed in distinct T cell populations, including CD3^+^CD4^+^CD28^+^, CD3^+^CD8^+^CD28^+^/CD28^-^, CD3^+^CD8^+^CD38^+^, CD3^+^CD8^+^HLA^-^DR^+^, and PD-1-expressing CD4^+^ and CD8^+^ T cells.

A separate staining panel was prepared for Treg cell detection: CD3 PB, CD4 APC-Cy7, CD25 PE, FOXP3 APC, and CD45 KO. Surface markers were stained first, followed by fixation and permeabilization for intracellular FOXP3 labeling. Treg frequency was calculated as the percentage of CD3^+^CD4^+^CD25^+^FOXP3^+^ cells within total CD4^+^ T lymphocytes.

### 2.7 Statistical analysis

Statistical analysis was performed by GraphPad Prism 9.5.1 software and SPSS 21.0 statistical software. Descriptive statistics were presented as mean ± standard deviation (SD) or range for continuous parametric data, and percentages for categorical data. The independent sample t test and Mann-Whitney test were employed to compare continuous data between the groups. Categorical data were analyzed using the Pearson’s chi-square test. Correlations were evaluated through Spearman tests. Multivariate analyses adjusted for age and sex were performed using multivariate logistic regression. The receiver operating characteristic (ROC) curve was used for diagnosability comparisons and the predictive effect was evaluated according to the area under the curve (AUC). P < 0.05 was considered statistically significant.

## 3 Results

### 3.1 Clinical data of the included population

A total of 123 patients with TED were enrolled in the current study, including 51 males and 72 females. The enrolled patients were divided into two groups based on the evaluation criteria of lacrimal gland enlargement: the NLG group consisting of 36 patients, and the ELG group consisting of 87 patients. The average age was younger in the ELG group as compared with that in the NLG group (45. 51 vs 51.22, P = 0.0141). However, there was no significant difference in the sex ratio between the two groups (P = 0.1589). Notably, a higher proportion of male patients was observed in the ELG group than in the NLG group (46.0% vs 30.5%). The average maximum area of the lacrimal gland in the oblique coronal position in the NLG and ELG groups was 0.79 and 1.27 cm^2^ respectively (P < 0.0001, 95% CI: 0.4100 to 0.5476). The baseline demographic characteristics of patients were presented in **Table 1**.

**Table 1:** Comparisons of clinical characteristics between the NLG and ELG groups in TED patients.

Characteristics	NLG	ELG	P-value
Number of cases	36	87	
Age at presentation, mean years (range)	51.22 (24-75)	45.51 (17-73)	0.0141*
Sex (female: male)	25: 11	47: 40	0.1589
Lacrimal gland area (cm2)	0.79 ± 0.13	1.27 ± 0.19	< 0.0001*

NLG, normal lacrimal gland; ELG, enlarged lacrimal gland. Lacrimal gland area: The average maximum area of the lacrimal gland in the oblique coronal position. Data are expressed as mean with range or cases. *P < 0.05.

### 3.2 Ocular evaluations

Out of all enrolled TED patients, 90 completed the comprehensive ocular evaluation (NLG: ELG = 25: 65) including the CAS, ocular proptosis, TBUT, and Schirmer I tests (**Table 2**). No significant differences were found in comparing disease activity based on the CAS assessment between the NLG and ELG groups (P = 0.6426). We further compared the TED severity between the NLG and ELG groups according to the EUGOGO standard (mild, moderate-to-severe, and sight-threatening). Pearson’s chi-square test showed no significant difference between the two groups (χ² = 0.6756, df = 2, P = 0.7136) (**Supplementary Table 1**). In addition, TED patients with enlarged lacrimal glands had a greater degree of proptosis compared with normal lacrimal glands (20.15 ± 2.67 mm vs 18.77 ± 2.41 mm, P = 0.0266). Subsequently, we compared the relationship between tear-associated parameters and lacrimal gland enlargement. First, TBUT less than 10 seconds was observed in over 90% of patients. Further comparison revealed that TBUT was significantly lower in the ELG group than in the NLG group (3.72 ± 2.34 s vs 5.22 ± 3.36 s, P = 0.0188). However, the Schirmer I test indicated that the tear secretion in the ELG group was significantly higher than that in the NLG group (16.02 ± 8.49 mm vs 12.07 ± 7.43 mm, P = 0.0443). Among patients in the ELG group, Schirmer I tests of 32 patients (49.2%) exceeded 15 mm, suggesting that these patients mainly presented with increased tear production. Collectively, these findings indicated that patients with enlarged lacrimal glands exhibited worse proptosis, unstable tear film, and excessive tearing.

**Table 2:** Comparisons of ocular features between the NLG and ELG groups in TED patients.

Characteristics	NLG (n = 25)	ELG (n = 65)	P-value
CAS (inactive: active)	15: 10	35: 30	0.6426
Exophthalmos (mm)	18.77 ± 2.41	20.15 ± 2.67	0.0266*
TBUT (s)	5.22 ± 3.36	3.72 ± 2.34	0.0188*
Schirmer I test (mm)	12.07 ± 7.43	16.02 ± 8.49	0.0443*

NLG, normal lacrimal gland; ELG, enlarged lacrimal gland; CAS, clinical activity score; TBUT, tear film break-up time. Data are expressed as mean with standard deviation (SD). *P < 0.05.

### 3.3 Thyroid function tests

The serum levels of thyroid-related hormones and autoantibodies of TED patients from the two groups were summarized in **Table 3**. The serum TG, TSH, FT3, FT4, TT3, and TT4 showed no significant difference between the ELG and NLG groups. It is well-established that the most frequently reported thyroid antibodies comprise TR-Ab, TG-Ab, and TPO-Ab (27). Despite no difference in the levels of TR-Ab and TG-Ab between the two groups (P = 0.0769, P = 0.3105, respectively), the average levels of TR-Ab and TG-Ab in the ELG group were over 3 times higher compared to those in the NLG group. Remarkably, the serum level of TPO-Ab was higher in patients in the ELG group than in the NLG group (P = 0.0166). Furthermore, Spearman correlation analysis confirmed a significant positive association between TPO-Ab levels and lacrimal gland cross-sectional area (r = 0.2814, P = 0.0027) (**Supplementary Figure 1a**). After adjusting for age and sex, TPO-Ab (OR = 1.1505, 95% CI: 1.0105–1.3098, P = 0.0342) remained independent risk factor for lacrimal gland enlargement (**Supplementary Table 1**). The findings collectively indicated that TPO-Ab was likely to exhibit the strongest association with lacrimal gland enlargement in TED among these thyroid-related autoantibodies.

**Table 3:** Comparisons of thyroid function tests between the NLG and ELG groups in TED patients.

Laboratory examinations	NLG (n = 30)	ELG (n = 82)	P-value
TG (ng/mL)	153.72 ± 211.46	196.18 ± 319.99	0.5017
TSH (μIU/mL)	1.28 ± 1.87	3.40 ± 9.01	0.2058
FT3 (pmol/L)	5.46 ± 1.26	6.56 ± 4.68	0.2062
FT4 (pmol/L)	11.09 ± 3.84	13.91 ± 10.78	0.1641
TT3 (nmol/L)	1.75 ± 0.64	2.11 ± 1.14	0.1086
TT4 (nmol/L)	106.36 ± 31.95	125.02 ± 58.97	0.1031
TR-Ab (IU/L)	9.45 ± 9.58	29.07 ± 59.74	0.0769
TG-Ab (IU/ml)	24.6 ± 112.23	85.56 ± 319.76	0.3105
TPO-Ab (IU/ml)	35.67 ± 93.11	183.1 ± 326.12	0.0166*

NLG, normal lacrimal gland; ELG, enlarged lacrimal gland; TG, thyroglobμlin; TSH, thyroid-stimμlating hormone; FT3, free triiodothyronine; FT4, free thyroxine; TT3, total triiodothyronine; TT4, total thyroxine; TR-Ab, TSH receptor antibody; TG-Ab, thyroglobμlin antibody; TPO-Ab, thyroid peroxidase antibody. Data are expressed as mean ± SD. *P < 0.05.

### 3.4 Serum cytokines

No statistical differences were detected in the levels of these six serum cytokines (IL-2, IL-4, IL-6, IL-10, TNF-α, and IFN-γ) between the NLG and ELG groups (**Table 4**).

**Table 4:** Comparisons of serum cytokines subsets between the NLG and ELG groups in TED patients.

Serum cytokines	NLG (n = 30)	ELG (n = 82)	P-value
IL-2 (pg/ml)	1.32 ± 0.69	1.24 ± 0.55	0.4978
IL-4 (pg/ml)	1.59 ± 0.79	1.39 ± 0.67	0.1874
IL-6 (pg/ml)	3.13 ± 1.52	3.43 ± 2.31	0.5138
IL-10 (pg/ml)	1.9 ± 0.82	1.64 ± 0.76	0.1167
TNF-α (pg/ml)	1.56 ± 0.67	1.44 ± 0.57	0.3761
IFN-γ (pg/ml)	1.73 ± 0.99	1.5 ± 0.68	0.1633

NLG, normal lacrimal gland; ELG, enlarged lacrimal gland; IL, interleukin; TNF-α, tumor necrosis factor-α; IFN, interferon-γ. Data are expressed as mean ± SD.

### 3.5 Peripheral lymphocyte subgroups and T cell subsets

Flow cytometry was performed to analyze peripheral lymphocyte subgroups in TED patients included in this study. As presented in **Table 5**, the absolute counts (595.56 ± 227.87 cells/ml vs 464.55 ± 155.22 cells/ml, P = 0.0469) and percentages (26.08 ± 6.85% vs 22.75 ± 7.10%, P = 0.0259) of CD8^+^ T cells were higher in TED patients in the ELG group than the NLG group. Accordingly, the ratio of CD4^+^/CD8^+^ T cells was lower in the ELG group as compared with the NLG group (1.74 ± 0.75 vs 2.13 ± 0.99, P = 0.0289). However, there were no obvious differences observed in the absolute counts and percentages of T cells, B cells, NK cells, and CD4^+^ T cells between the two groups.

**Table 5:** Comparisons of lymphocyte subgroups between the NLG and ELG groups in TED patients.

Lymphocyte subsets	NLG (n = 30)	ELG (n = 82)	P-value
CD3+CD19-T cell (%)	68.75 ± 9.25	71.43 ± 7.71	0.1249
CD3+CD19-T cell (cells/μl)	1302.02 ± 438.67	1610.05 ± 698.09	0.1200
CD3-CD19+B cell (%)	16.96 ± 6.42	15.8 ± 6.57	0.4056
CD3-CD19+B cell (cells/μl)	359.76 ± 211.86	389 ± 267.84	0.7079
CD3+CD4+T cell (%)	42.6 ± 9.31	40.95 ± 8.72	0.3872
CD3+CD4+T cell (cells/μl)	791.35 ± 350.7	916.8 ± 486.62	0.3696
CD3+CD8+T cell (%)	22.75 ± 7.10	26.08 ± 6.85	0.0259*
CD3+CD8+T cell (cells/μl)	464.55 ± 155.22	595.56 ± 227.87	0.0469*
CD3-CD16+CD56+NK cell (%)	13.67 ± 9.08	12.27 ± 7.16	0.3944
CD3-CD16+CD56+NK cell (cells/μl)	262.3 ± 176.59	255.42 ± 131.94	0.8781
CD3+CD4+/CD3+CD8+	2.13 ± 0.99	1.74 ± 0.75	0.0289*

NLG, normal lacrimal gland; ELG, enlarged lacrimal gland; Data are expressed as mean ± SD. *P < 0.05.

Nevertheless, Spearman correlation analysis confirmed that the proportion of CD8^+^ T cells did not significantly correlate with the quantitative lacrimal gland area (r = 0.0736, P = 0.4405) (**Supplementary Figure 1b**). Furthermore, after adjusting for age and sex via multivariate analysis, the association between CD8^+^ T cells and ELG lost its statistical significance (OR 1.0607, 95% CI 0.9890-1.1375, P = 0.0989), suggesting that this peripheral immune feature might be partially influenced or confounded by demographic factors.

Relevantly, we conducted further analysis on functional subsets of T lymphocyte inhibitory and activation (**Table 6**). Consistently, the ELG group exhibited a significant increase in the proportion of cytotoxic CD8^+^ T lymphocytes (27.99 ± 8.17% vs 22.60 ± 6.40%, P = 0.0242), along with a decreased ratio of CD4^+^/CD8^+^ T cells (1.49 ± 0.58 vs 1.98 ± 0.98, P = 0.0322), compared to the NLG group. Additionally, we observed a significant decrease in the proportion of PD-1^+^CD8^+^ T cells within the ELG group (2.89 ± 2.35 vs 5.00 ± 3.00, P = 0.0089), while no disparities were detected among other subsets of T lymphocytes.

**Table 6:** Comparisons of T cell subsets between the NLG and ELG groups in TED patients.

T cell subsets	NLG (n=16)	ELG (n=35)	P-value
Helper T lymphocyte functional subsets (%) (CD3^+^CD4^+^CD28^+^/CD3^+^CD4^+^)	95.77 ± 5.25	95.98 ± 6.02	0.9028
Cytotoxic T lymphocyte functional subsets (%) (CD3^+^CD8^+^CD28^+^/CD3^+^CD8^+^)	72.93 ± 15.91	71.34 ± 18.84	0.7714
Suppressor T lymphocyte (%) (CD3^+^CD8^+^CD28^-^/LYM)	6.19 ± 3.86	8.50 ± 6.65	0.2027
Activated T lymphocyte (%) (CD3^+^HLA-DR^+^/LYM)	14.06 ± 6.24	13.89 ± 6.22	0.9278
Cytotoxic T lymphocyte activated subsets (%) (CD3^+^CD8^+^CD38^+^/CD3^+^CD8^+^)	44.66 ± 16.88	43.79 ± 19.88	0.8805
Cytotoxic T lymphocyte activated subsets (%) (CD3^+^CD8^+^HLA-DR^+^/CD3^+^CD8^+^)	33.08 ± 17.14	28.79 ± 14.71	0.3632
PD-1 expression in Helper T lymphocyte (%) (CD3^+^CD4^+^PD1^+^/CD3^+^CD4^+^)	3.32 ± 1.55	3.19 ± 2.55	0.8583
PD-1 expression in cytotoxic T lymphocyte (%) (CD3^+^CD8^+^PD1^+^/CD3^+^CD8^+^)	5.00 ± 3.00	2.89 ± 2.35	0.0089*
Helper T lymphocyte (%) (CD3^+^CD4^+^)	40.11 ± 9.49	38.44 ± 9.59	0.5647
Cytotoxic T lymphocyte (%) (CD3^+^CD8^+^)	22.60 ± 6.40	27.99 ± 8.17	0.0242*
CD3^+^CD4^+^/CD3^+^CD8^+^	1.98 ± 0.98	1.49 ± 0.58	0.0322*
Naive CD4^+^T cell (%) (CD3^+^CD4^+^CD45RA^+^/CD3^+^CD4^+^)	38.41 ± 12.40	37.12 ± 15.65	0.7730
Memory CD4^+^T cell (%) (CD3^+^CD4^+^CD45RO^+^/CD3^+^CD4^+^)	61.59 ± 12.40	62.88 ± 15.65	0.7721

NLG, normal lacrimal gland; ELG, enlarged lacrimal gland; Data are expressed as mean ± SD. *P < 0.05.

Overall, increased CD8^+^ T cells and decreased exhausted PD-1^+^CD8^+^ T cells were positively correlated with enlarged lacrimal glands in TED without linear correlation with quantitative lacrimal gland area. Further analysis confirmed that age and sex interfered with the observed association of CD8^+^ T cells and lacrimal gland enlargement.

### 3.6 Treg cells

**Figure 2:**
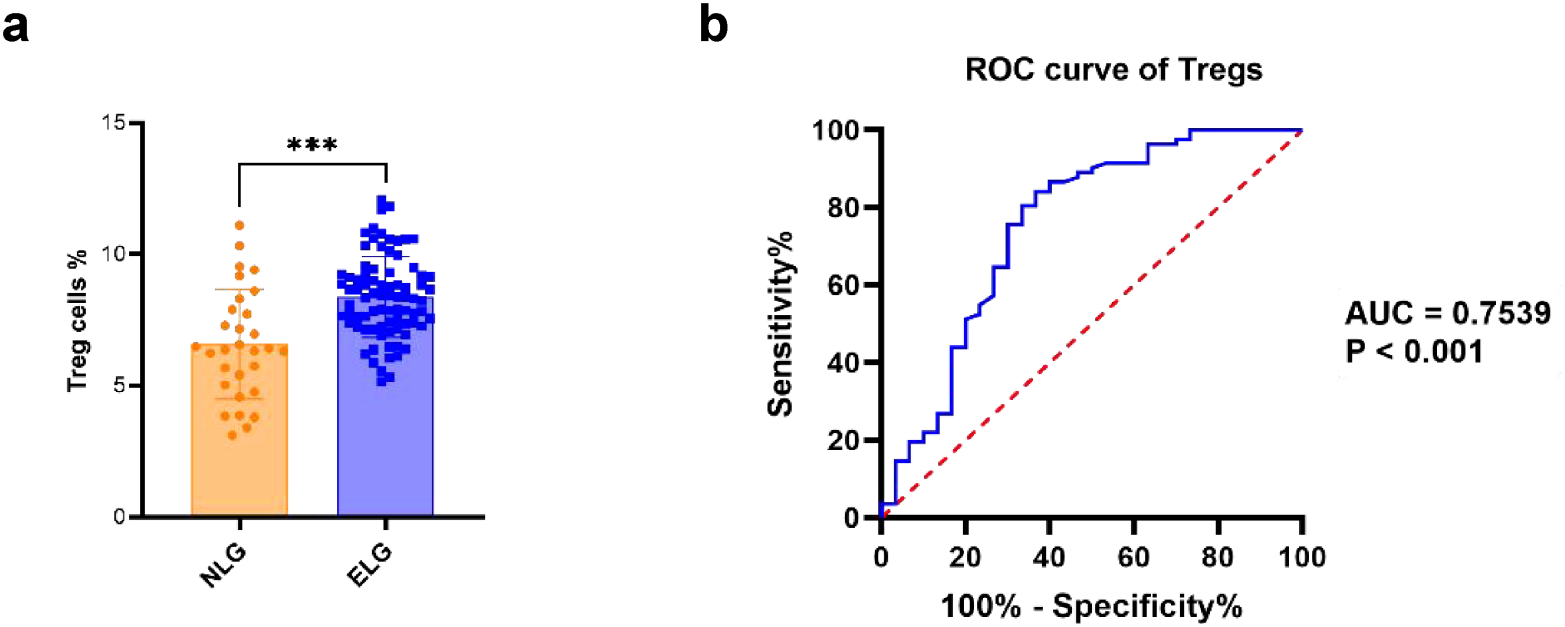
The associations between Treg cells and lacrimal gland enlargement. **(a)** Comparisons of the percentage of Treg cells between the NLG (n = 30) and ELG (n = 82) groups. Data are expressed as mean ± S.D. **(b)** The ROC curve test indicated the predictive performance of serum Treg cells in lacrimal gland enlargement in patients with TED. ns = not significant; ***P < 0.001; ROC, receiver operator characteristic; AUC, area under the curve.

Regulatory T (Treg) cells, a subset of CD4^+^ T lymphocytes expressing high levels of CD25 and the transcription factor forkhead box P3 (FOXP3), serve as negative regulators of inflammation and play an important role in the prevention of autoimmune disease (28). Our results showed that the percentage of Treg cells was remarkably elevated in the ELG group, compared to the NLG group (P < 0.001, 95% CI: 1.075 to 2.511) (**Figure 2a**). Furthermore, the ROC curve analysis indicated good predictive performance of serum Treg level in lacrimal gland involvement in patients with TED (AUC = 0.7539, P < 0.001, 95% CI: 0.6394 to 0.8683) (**Figure 2b**). Furthermore, the percentage of Treg cells exhibited a significant positive correlation with the lacrimal gland area, as confirmed by Spearman correlation analysis (r = 0.3041, P = 0.0011) (**Supplementary Figure 1c**). Treg also showed a significant association after adjustment (OR 1.8084, 95% CI 1.3097–2.4970, P = 0.0003) (**Supplementary Table 2**). Together, the increase in Treg cells not only correlated closely with physical lacrimal gland enlargement but also served as an indicator of lacrimal gland involvement in TED.

## 4 Discussion

The ocular phenotypes associated with lacrimal gland enlargement in TED, along with the underlying pathogenesis and potential treatments, remain to be elucidated. In this study, 87 of 123 TED patients (70.7%) exhibited bilateral enlarged lacrimal glands. We conducted an in-depth investigation into the diverse spectrum of ocular manifestations and the array of immunological abnormalities detected in the peripheral blood of TED patients with lacrimal gland enlargement, aiming to shed light on the etiological factors.

Relevant ocular symptoms between the NLG and ELG groups were compared. First, no statistically significant differences in TED activity and severity were found between the two groups based on CAS (**Table 2**) and EUGOGO grading (**Supplementary Table 1**). TED patients with lacrimal gland enlargement had worse proptosis, shorter TBUT, and higher Schirmer I test values, compared with those with normal lacrimal glands (**Table 2**). Indeed, a substantial proportion of patients in the ELG group exhibited a Schirmer I test result exceeding 15 mm, indicating an increased likelihood of experiencing symptoms related to tearing. We hypothesized that lacrimal gland enlargement in TED was associated with symptoms of excessive tearing due to inflammatory response, whereas lacrimal gland hypofunction was related to tear film instability and dry eye (29). Consequently, enlarged lacrimal glands may manifest symptoms associated with both dry eye and tearing concurrently, underscoring the significance of enhancing tear film stability rather than quantity in addressing TED-induced ocular surface manifestations.

Subsequently, we investigated the correlation between thyroid function and lacrimal gland enlargement. There were no differences in thyroid hormone levels; the main variations were observed in the thyroid-associated antibodies (**Table 3**). We identified that the level of TPO-Ab in TED patients with enlarged lacrimal glands was significantly higher than in patients with normal lacrimal glands. A systematic review concluded that the relationship between TPO-Ab and TED remained unclear and controversial (30). Several studies demonstrated there was a positive correlation between elevated TPO-Ab levels and ocular manifestations in children with TED (31, 32). Correlation analysis revealed that serum TPO-Ab levels were positively correlated with lacrimal gland area. Multivariate logistic regression analysis (**Supplementary Table 2**) adjusted for age and sex confirmed that TPO-Ab was an independent factor associated with lacrimal gland enlargement in TED, suggesting that TPO-Ab may play a crucial role in both the hypertrophy and dysfunction of the lacrimal gland.

Given the intricate immune network within the lacrimal gland, the precise associations between cytokines, T cell subsets, and lacrimal gland enlargement in TED remain elusive (33). Previous studies have indicated a significant increase in cytokine levels among patients with hyperthyroidism (3). However, no notable disparities in serum cytokines were observed between the NLG and ELG groups (**Table 4**). This suggests that the immune dysregulation driving lacrimal gland enlargement in TED is predominantly localized to the orbital microenvironment rather than being a systemic response. Immune dysregulation in TED occurs predominantly locally in the orbit rather than as a systemic immune abnormality (34). T cells secrete cytokines *in situ* within the orbit to mediate remodeling of the orbital tissues including the lacrimal gland (35). Future studies analyzing local biospecimens, such as tear fluid, are warranted to confirm this compartmentalized inflammation (36).

Given the lacrimal gland’s status as an extraorbital organ endowed with a highly vascularized network, our investigation primarily focused on establishing a relationship between lymphocyte classification in peripheral blood and lacrimal gland enlargement (**Table 5**) (37). In the ELG group, we observed a significant increase in the proportion of cytotoxic CD8^+^ T lymphocytes in the peripheral blood of TED patients, accompanied by a significant decrease in the corresponding CD4^+^/CD8^+^ T cell ratio. Further analysis revealed that PD-1^+^CD8^+^ T lymphocytes were significantly down-regulated in the ELG group (**Table 6**). PD-1 has been identified to exert inhibitory effects on T-cell activation (38). Downregulation of PD−1 on CD8^+^ T cells relieves inhibitory signaling, reduces negative feedback, and enhances the cytotoxic potential of these cells. This phenotype supports a more activated, pro−inflammatory state within the lacrimal gland microenvironment, which may promote local inflammation, edema, and subsequent glandular enlargement. As reported by Frebel et al., PD−1 deficiency enables unrestricted, antigen−specific cytotoxicity toward structural cells via perforin−dependent pathways, thereby impairing vascular integrity and inducing tissue edema (39). Collectively, these observations suggest that defective PD−1−mediated immune regulation contributes to lacrimal gland involvement in TED. Thus, an increase of CD8^+^ T cells and decreased PD-1^+^CD8^+^ T cells implied that the aberrantly hyperactivated and increased CD8^+^ T cells presented in the peripheral blood infiltrated into the lacrimal gland. However, after adjusting for age and sex in multivariate logistic regression, CD8^+^ T cell proportion was no longer an independent predictor of lacrimal gland enlargement, indicating that the initial association was confounded by demographic factors.

Treg cells, a distinct subset of CD4^+^ T lymphocytes, play a crucial role as negative regulators in the context of inflammatory diseases (40). Relevant studies have previously demonstrated that the proportion of Treg cells is typically diminished in patients with GD compared to normal controls (41). In our study, Treg cells were significantly increased in TED patients with lacrimal gland enlargement with high diagnostic value (**Figures 2a, b**). Given the immune regulative role of Treg cells, our results implied a possible endogenous protective role of immune regulation through the negative feedback loop of Treg cell activation. Correlation analysis (**Supplementary Figure 1c**) identified a positive correlation between Treg proportion and lacrimal gland area; after adjustment for age and sex, multivariate logistic regression (**Supplementary Table 2**) confirmed Treg proportion as an independent factor positively associated with lacrimal gland enlargement in TED.

The limitation of this study should be acknowledged. Conventional MRI cannot non-invasively differentiate between active interstitial edema, acute inflammatory cell infiltration, and chronic fibrosis within the lacrimal gland. Future studies utilizing advanced quantitative imaging sequences, such as T2/T1 mapping, extracellular volume (ECV) fraction, and radiomics, are required to achieve precise non-invasive imaging-pathology matching and further clarify the exact histopathological transitions associated with these peripheral immune changes (42, 43).

In summary, our results demonstrated that TED patients with enlarged lacrimal glands exhibited worse proptosis, unstable tear film, and excessive tearing. In particular, we identified TPO-Ab as the thyroid-associated antibodies most associated with lacrimal gland enlargement in TED. Immunological evidence suggested that Treg cells were positively correlated with lacrimal gland enlargement in TED. Further research is needed to investigate linking these immune factors to lacrimal gland enlargement in TED.

## Data Availability

The raw data supporting the conclusions of this article will be made available by the authors, without undue reservation.
