# Supplementary figures and images for: Ocular manifestations and immunological profiles of thyroid eye disease with lacrimal gland enlargement
Source: Front Endocrinol (Lausanne). 2026 Jul 15;17:1861245. doi: 10.3389/fendo.2026.1861245 (PMC13414220; doi:10.3389/fendo.2026.1861245)

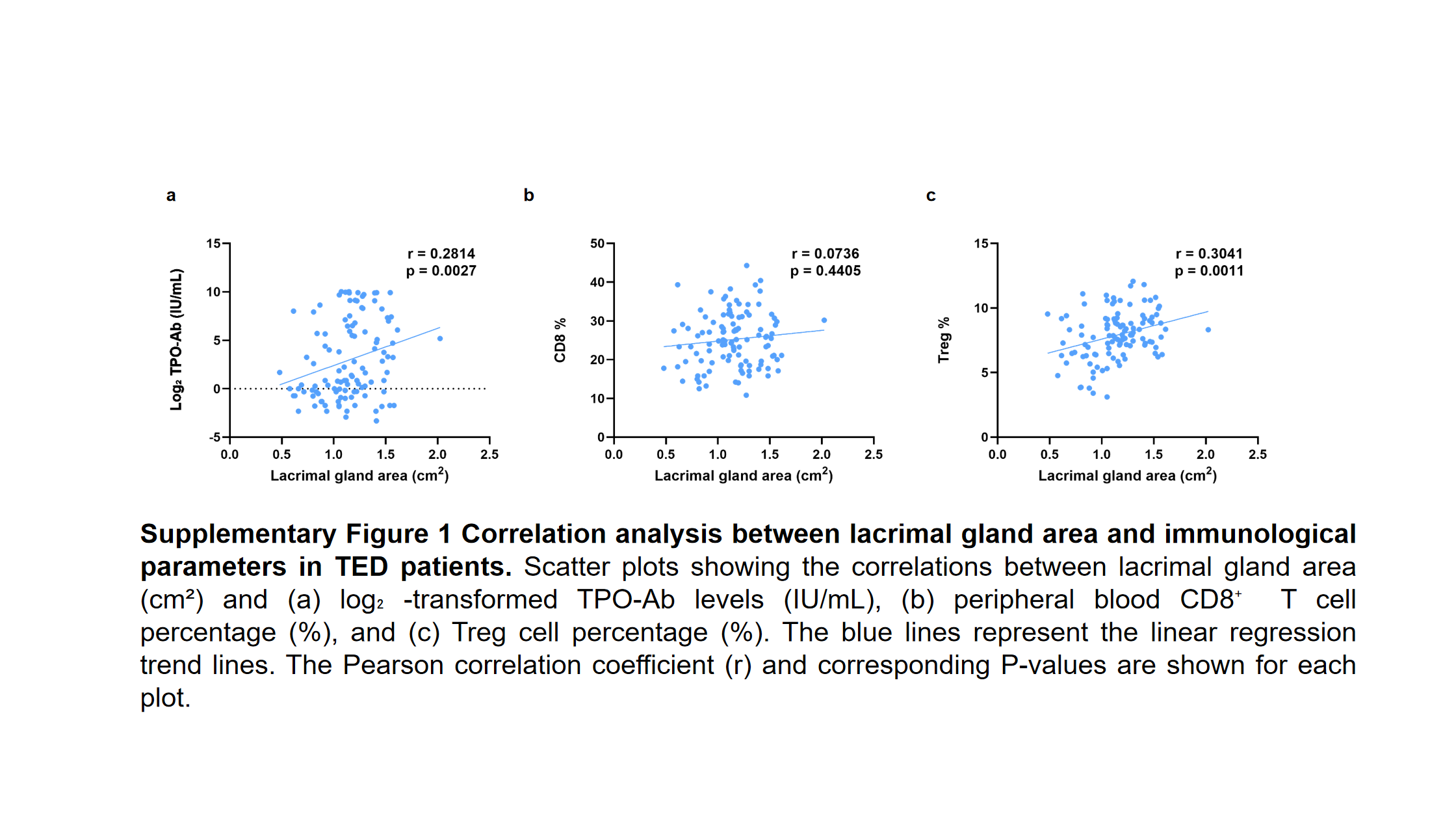

Supplement: Supplementary file 1 [file Image1.tif]
