# Supplementary material for: Ocular manifestations and immunological profiles of thyroid eye disease with lacrimal gland enlargement
Source: Front Endocrinol (Lausanne). 2026 Jul 15;17:1861245. doi: 10.3389/fendo.2026.1861245 (PMC13414220; doi:10.3389/fendo.2026.1861245)
Supplement: Supplementary file 2 [file Table1.docx]

**Supplementary Table 1 |** Comparisons of EUGOGO grade between the NLG and ELG groups in TED patients.

| **EUGOGO** | **NLG (n = 25)** | **ELG (n = 65)** | **χ²** | **P-value^a^** |
| --- | --- | --- | --- | --- |
| Mild | 5 (20.0%) | 9 (13.8%) | 0.6756 | 0.7136 |
| Moderate-to-Severe | 16 (64.0%) | 47 (72.3%) |  |  |
| Sight-threatening | 4 (16.0%) | 9 (13.8%) |  |  |

^a^ Pearson chi-square test; European Group on Graves’ Orbitopathy; NLG = normal lacrimal gland; ELG = enlarged lacrimal gland.

**Supplementary Table 2** | Multivariate logistic regression analysis for factors associated with lacrimal gland enlargement in TED, adjusted for Age and Sex.

| **Variables** | **OR (95% CI)** | **Z-value** | **P-value** |
| --- | --- | --- | --- |
| Log_2_(TPO-Ab) | 1.1505 (1.0105-1.3098) | 2.1179 | 0.0342* |
| CD8 | 1.0607 (0.9890-1.1375) | 1.6502 | 0.0989 |
| Treg | 1.8084(1.3097-2.4970) | 3.599 | 0.0003* |

OR = odds ratio; CI = confidence interval; *P < 0.05.
